# The effect of mindfulness-based couple-centered intervention in parents following fetal abnormalities requiring pregnancy termination: study protocol for a randomized controlled trial

**DOI:** 10.1186/s12884-025-08138-2

**Published:** 2025-10-02

**Authors:** Shiwen Sun, Jialu Qian, Yaping Sun, Chunxiao Hu, Xiaojuan Zeng, Ju Ma, Ying Yao, Kaiying Wang, Xiaoyan Yu

**Affiliations:** 1 Department of nursing, Women’s Hospital School of Medicine, Zhejiang University, Hangzhou, Zhejiang China; 2 Nanjing University of Chinese Medicine, School of Nursing, Nanjing, Jiangsu China

## Abstract

**Background:**

Termination of pregnancy for fetal abnormalities (TOPFA) represents a significant global healthcare challenge. In China, the occurrence of fetal abnormalities is notably high. TOPFA is a devastating traumatic event for parents and families. Parents frequently encounter psychological issues and negative emotions such as depression, anxiety, prolonged grief, feelings of inadequacy, persistent guilt, and symptoms of post-traumatic stress disorder (PTSD).

**Methods/design:**

This prospective, randomized controlled trial aims to assess the feasibility and impact of a mindfulness-based, couple-centered intervention (MBCI) on anxiety, depression, mindfulness level, and family support among bereaved parents. The study will enroll 124 couples experiencing TOPFA. Participants will be randomly assigned to either the intervention group (receiving MBCI in addition to routine psychological care) or the control group (receiving only routine psychological care). Data will be collected at three time points: baseline, post-intervention and 42 days postpartum. Additionally, participants are expected to complete a weekly mindfulness exercise feedback form. Semi-structured, in-depth interviews with the intervention group will be conducted post-intervention and 42 days postpartum.

**Discussion:**

This RCT aims to investigate the effectiveness of a couple-centered mindfulness intervention on the psychological well-being of parents experiencing TOPFA. If effective, this intervention could serve as a complement to routine psychological care, aiding in the physical and mental recovery of women and their families.

## Introduction

Fetal abnormalities encompass congenital malformations and stillbirths. In 2017, worldwide, 280,000 newborns died within 28 days of birth from congenital malformations, 14,000 of which were in China [1]. According to 2023 WHO data, approximately 1.9 million stillbirths and 2.3 million neonatal deaths occur globally each year [2].

**Fig. 1:**
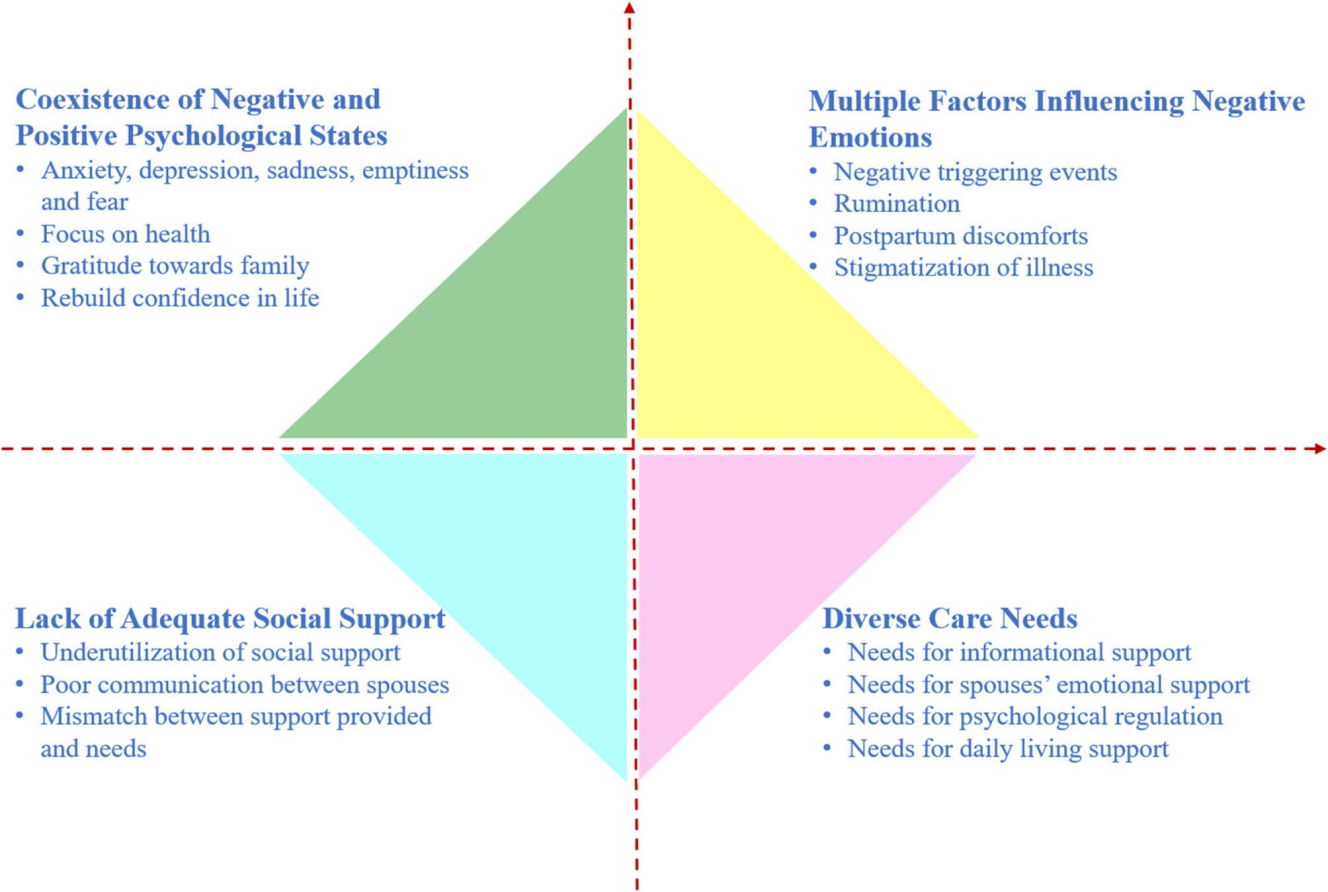
Psychological Experience of Women with TOPFA during Postpartum Period

The occurrence of sudden fetal anomalies or stillbirth during pregnancy is a significant traumatic event for pregnant women and their families, leading to intense and prolonged grief and destructive psychosocial issues [3, 4]. Approximately 80% of women who experience perinatal loss have symptoms of grief or bereavement [5], about 50% of women experience grief symptoms that last longer than 4 years [6], and often accompanied by comorbidities such as post-traumatic stress disorder (PTSD), anxiety, depression, sleep disturbances, and eating disorders [6, 7]. Psychological surveys of parents who underwent terminations of pregnancy for fetal abnormalities (TOPFA) showed that the prevalence of anxiety was 24.3% for mothers and 19.5% for fathers, depression was 50.3% for mothers and 24.9% for fathers, and PTSD was 21.3% for mothers and 20.1% for fathers, with significant correlations between anxiety, depression, and PTSD levels for both mothers and fathers [8, 9], indicating that both mothers and fathers experience significant PTSD, anxiety, and depression, and their negative emotions affect each other. Furthermore, stillbirth is an important risk factor for anxiety and depression during subsequent pregnancies and the postpartum period [10, 11]. Therefore, effective psychological interventions are urgently needed to provide synchronous interventions for both mothers and fathers [8, 9]. Qualitative interviews with mothers who experienced TOPFA during the postpartum period revealed that negative psychological experiences coexist with positive psychological experiences in this population, and their caregiving needs exhibit diverse characteristics [12], as shown in Fig. 1.

Currently, psychological care for parents undergoing TOPFA primarily consists of perinatal grief counseling, psychological counseling and treatment, and support from family, peers, groups, and associations [13–17]. The Swedish National Infant Foundation (SNIF) established a “self-help” group support model, offering a platform for parents to communicate and express their grief, thereby aiding them in adjusting better to their loss [18] Kersting et al. [19] conducted a five-week internet-based cognitive-behavioral intervention that effectively reduced symptoms of grief, depression, and PTSD in bereaved mothers. In the UK, charitable organizations like Antenatal Results and Choices (ARC) [20] and Stillbirth & Neonatal Death Society (Sands) offer social support and long-term follow-up for this group.

Mindfulness intervention, a novel psychological therapy, is widely used internationally for preventing or treating various psychological issues. Scholars have also applied mindfulness intervention to the prevention and treatment of anxiety and depression among pregnant and postpartum women. Lisa Roberts et al. [21] conducted a five-week localized mindfulness intervention for mothers who experienced stillbirth, showing that it reduced symptoms of perinatal grief, anxiety, and depression. Jennifer Huberty et al. [22] conducted a 12-week online mindfulness yoga intervention for mothers who experienced stillbirth, which effectively alleviated symptoms of PTSD, grief, and depression. Thieleman and Cacciatore [23] explored a grief-focused mindfulness-based retreat’s effectiveness on psychological distress and well-being in bereaved parents, finding it decreased trauma responses and distress. Chen et al. [24] and Zeng et al. [25] conducted mindfulness-based intervention studies focusing on four intensive short-term mindfulness training sessions during hospitalization, complemented by three weeks of home self-practice after discharge. These interventions effectively increased mindfulness levels and alleviated anxiety and depression in mothers.

Fathers experiencing TOPFA also indirectly suffer from the grief and psychological trauma associated with TOPFA. Negative psychological effects mutually influence both mothers and fathers [9], underscoring the necessity of including fathers in psychological interventions to promote synchronous recovery for both parents experiencing TOPFA. However, previous psychological interventions rarely included fathers, overlooking the importance of paternal psychology and its impact on maternal psychological health. Therefore, this study included both mothers and fathers who experienced TOPFA as research subjects simultaneously. This study aims to assess the feasibility and effects of a mindfulness-based, couple-centered intervention (MBCI) on anxiety, depression, mindfulness levels, and family support among bereaved parents.

## Methods

### Study design

This trial is randomized, involving parallel groups, and is controlled. The trial has been registered with the Chinese Clinical Trial Registry (ChiCTR2100054164). The study protocol follows the Standard Protocol Items: Recommendations for Interventional Trials (SPIRIT) checklist.

## Setting and recruitment

**Fig. 2:**
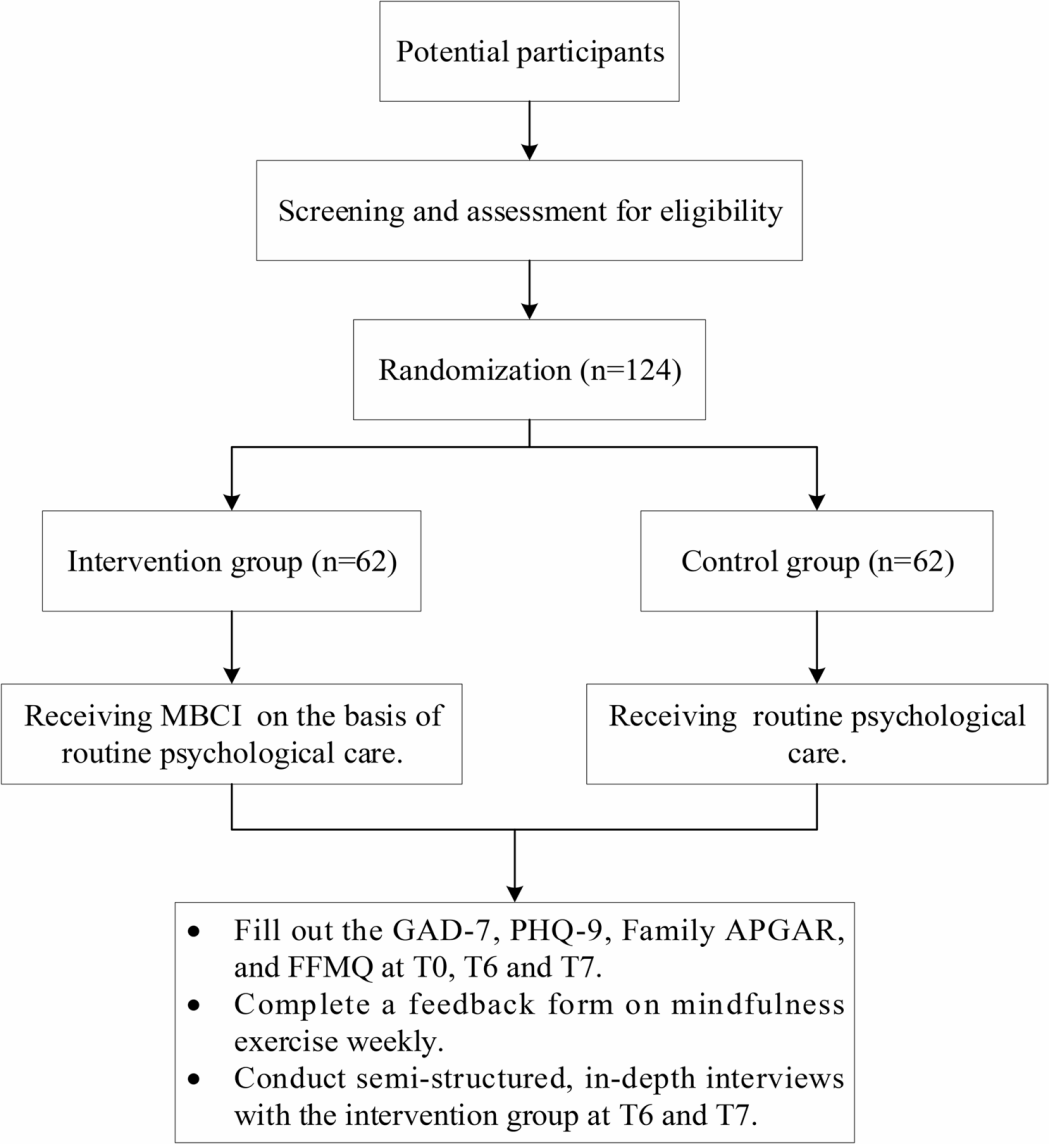
Flow diagram of enrollment, interventions, and assessment. T0: Baseline; T6: Post-intervention; T7: 42 days postpartum

Screening and enrollment of participants will occur on the admission day. This study conducted at the Women’s Hospital School of Medicine, Zhejiang University. This study aims to enroll 124 couples experiencing TOPFA, with 62 couples in both the intervention and control groups. The study’s recruitment phase was initiated on March 21, 2022, and the data collection process is projected to be finalized by October 31, 2025. The study’s flow chart is presented in Fig. 2.

## Eligibility criteria

The inclusion criteria include: a diagnosis of fetal abnormality confirmed by Zhejiang Provincial Prenatal Diagnosis Center and a decision to terminate the pregnancy due to the fetal abnormality after > 14 gestational weeks. Couples must be over 18 years old, have no prior participation in mindfulness-related psychological interventions or other scientific research, be proficient in using WeChat, willing to accept online interventions via WeChat, and both spouses must voluntarily participate and provide signed informed consent. The exclusion criteria include: a history of psychosomatic diseases; intellectual disabilities, illiteracy, or an inability to understand the intervention and assessment content; development of severe psychosomatic issues during the study, preventing participation; voluntary withdrawal or loss of contact during the study; incomplete research questionnaires or missing items.

## Randomization

After signing the informed consent, participants will be randomly assigned to obstetric wards 2 through 7. To prevent cross-contamination that could impact intervention effectiveness, those in wards 2 to 4 will be assigned to the intervention group, and those in wards 5 to 7 will be part of the control group. The intervention group will receive MBCI in addition to routine psychological care, whereas the control group will only receive routine psychological care.

## Intervention

### Framework of MBCI

**Fig. 3:**
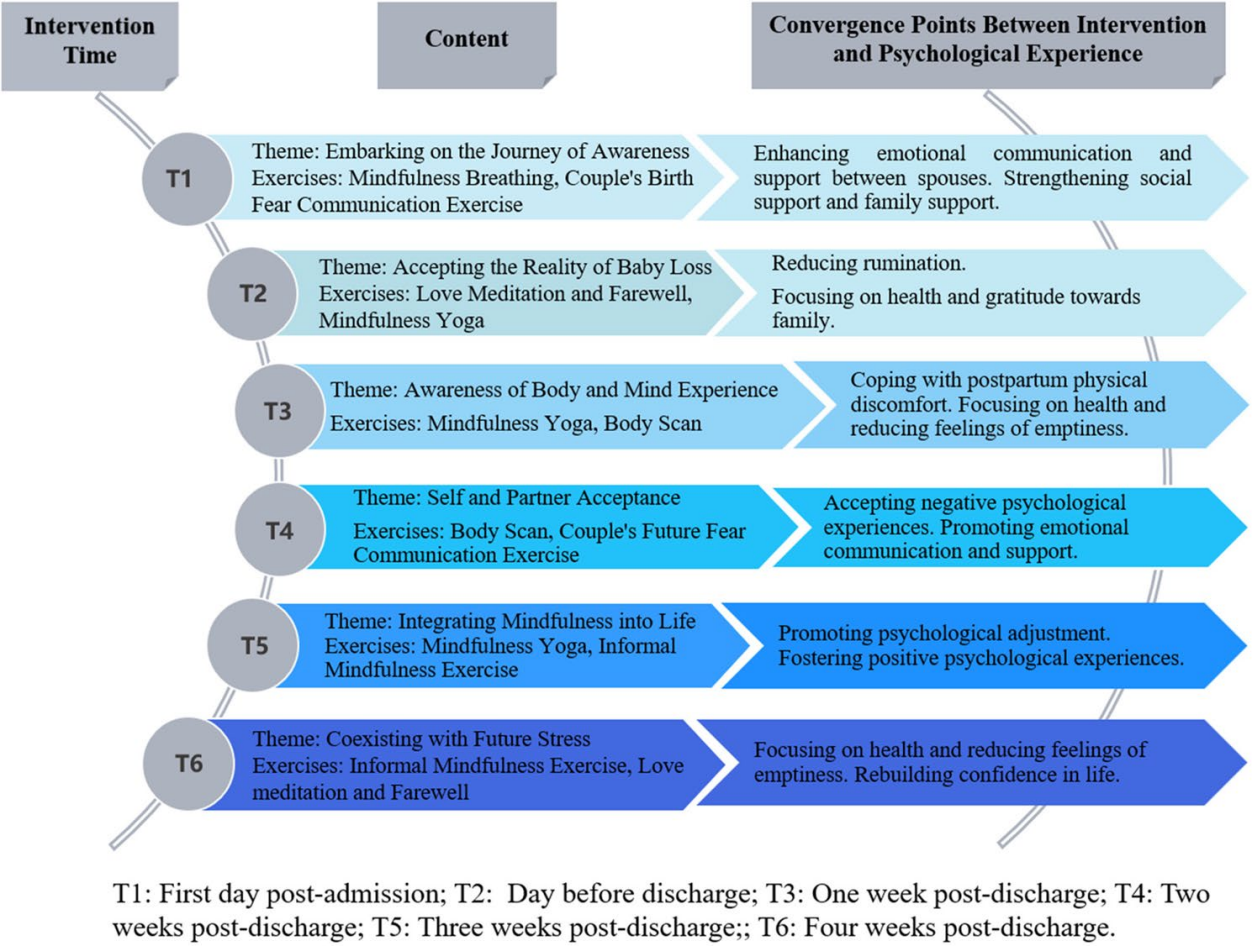
Framework diagram for MBCI

The MBCI framework is based on mindfulness attention-acceptance theory, incorporating techniques from Mindfulness-Based Stress Reduction (MBSR) and Mindfulness-Based Childbirth and Parenting (MBCP). It also integrates findings from previous research and literature on the psychology of parents who have experienced TOPFA. Researchers, psychological counselors, and mindfulness trainers have developed a preliminary framework diagram for MBCI (Fig. 3).

#### Implementation of MBCI

##### Intervention content

Six mindfulness intervention sessions will be conducted, with themes progressing sequentially. Each session includes four modules: A - Theoretical Explanation; B - Mindfulness Exercises; C - Couples’ Communication and Sharing; D - Homework Assignment. Table 1 details the specific contents.

**Table 1:** Content of mindfulness-based for couple-centered intervention

Theme	Content	Homework
Session 1: Embarking on the Journey of Awareness	**Theoretical Explanation**: explaining the origin, connotation, functions, and practice techniques of mindfulness. **Mindfulness Exercises**: ①Mindfulness Breathing: fostering continuous awareness of the present through breathing, cultivating a non-judgmental, open, and accepting mindset. ②Couple’s Birth Fear Communication Exercise: aiming to reduce fear of induced labor and enhance mutual understanding and support. **Communication and Sharing**: couples discuss and share their experiences with the mindfulness exercises.	①Practicing mindfulness breathing once a day ②Conducting a couple’s communication exercise, allowing the couple to choose the topic.
Session 2: Accepting the Reality of Baby Loss	**Theoretical Explanation**: explaining the role and methods of love meditation and farewell, alongside mindfulness yoga. **Mindfulness Exercises**: ①Love Meditation and Farewell: involves sending loving blessings to family and friends, offering blessings to the lost fetus, and bidding farewell inwardly. ②Mindfulness Yoga: pay attention to bodily sensations and emotional changes during stretching exercises, maintaining an open, accepting, and non-judgmental attitude. **Communication and sharing**: same as the previous session.	①Engaging in mindfulness yoga exercise once daily. ②Participating in one couple’s love meditation exercise, expressing gratitude for mutual support and companionship.
Session 3: Awareness of Body and Mind Experience	**Theoretical Explanation**: delving into the meaning and significance of attention in mindfulness. **Mindfulness Exercises**: ①Mindfulness Yoga: same as the previous session. ②Body Scan: sequentially experience and sense body sensations with a non-judgmental, curious, and open attitude. **Communication and sharing**: same as the previous session.	①Engaging in mindfulness yoga exercise once daily. ②Conducting one body scan exercise
Session 4: Self and Partner Acceptance	**Theoretical Explanation**: understanding the meaning and importance of an acceptance attitude in mindfulness. **Mindfulness Exercises**: ①Body Scan: identical to the previous session. ②Couple’s Future Fear Communication Exercise: encourages couples to understand and accept each other’s future fears, fostering mutual support. **Communication and sharing**: identical to the previous session.	①Engaging in one body scan exercise daily. ②Participating in a couple’s communication exercise with topics chosen by the couple.
Session 5: Integrating Mindfulness into Life	**Theoretical Explanation**: explaining the meaning, effects, and methods of informal mindfulness practice. **Mindfulness Exercises**: **①**Mindfulness Yoga: same as the previous session. ②Informal Mindfulness Exercises: using raisin meditation as an example. **Communication and sharing**: same as the previous session.	①Engaging in mindfulness yoga practice once daily. ②Engaging in informal mindfulness practice once daily.
Session 6: Coexisting with Future Stress	**Theoretical Explanation**: summarizing and reviewing the theoretical content from the previous five sessions. **Mindfulness Exercises**: ①Informal Mindfulness Exercises: content identical to the previous session. ②Love Meditation and Farewell: repeats the loving-kindness meditation to bid farewell to this special experience and offer future blessings. **Communication and sharing**: same as the previous session.	①Continuing to practice informal mindfulness. ② Using mindfulness to manage future stress.

## Intervention format

The first two sessions will focus primarily on face-to-face intervention on-site, with online intervention as a supplement, whereas the remaining four sessions will be online. Online interventions will be conducted via the WeChat platform, where mindfulness training audios are available. Participants can practice using these audios.

### Intervention duration

Pregnancy mindfulness interventions typically include 6–8 sessions, with each session lasting 0.5–2 h [26]. This provides a basis for determining the duration of mindfulness intervention in this study. Taking into account the Chinese tradition of 30-day postpartum confinement, the intervention period extends from hospitalization for induced abortion to four weeks postpartum, with sessions lasting 1.5 to 2 h each.

### Routine psychological care

The control group receives routine psychological care, graded care based on the psychological assessment of women upon admission [27]. Specific Content: (a) women in good psychological states receive general psychological comfort from nurses, explanations about fetal abnormalities, induced labor, postpartum care, and preparation for future pregnancies; (b) for women with mild to moderate psychological issues, nurses develop a psychological counseling plan involving family members; (c) women with severe psychological disorders require counseling under a psychological expert’s guidance or are advised to seek psychiatric psychotherapy. Researchers will offer MBCI and related guidance to participants at the study’s end, considering ethical concerns.

### Intervention fidelity

A senior psychological counselor supervises the entire intervention process. The mindfulness intervention in this study is conducted by a psychological counselor who had received systematic mindfulness training. The mindfulness exercise audios are recorded by this counselor. Participants engage in mindfulness exercises through the WeChat platform and complete feedback forms there. Researchers promptly analyze mindfulness exercise feedback, address issues, and communicate with participants online, encouraging daily engagement.

### Measurement instruments

The personal information form includes two sections: demographic characteristics (age, education level, occupation, religious beliefs, income level, personality traits, marital status, living conditions, living arrangements) and obstetric characteristics (pregnancy and childbirth history, history of abnormal pregnancies, gestational age, pregnancy intentions, fertilization method, fetal abnormality diagnosis).

Mindfulness exercise feedback form created by researchers will be used to gather insights into participants’ mindfulness exercise experiences. It covers exercise duration, challenges faced, feelings during the exercise, satisfaction with the intervention (content, methodology, duration), and any suggestions for improvement.

The Generalized Anxiety Disorder Scale (GAD-7) assesses anxiety severity and functional impairment over the past two weeks with 7 items, each scored from 0 to 3. Scores range from 0 to 21, where 0–4 indicate no anxiety, 5–9 mild anxiety, 10–14 moderate anxiety, and ≥ 15 severe anxiety. The Chinese version is reliable and valid, with a Cronbach’s α of 0.92 [28].

The Patient Health Questionnaire (PHQ-9), a 9-item, 4-point scale, measures maternal depression. The PHQ-9, known for its simplicity and sensitivity, is commonly used to assess antenatal depression. Total scores, reflecting the sum of all items, indicate the severity of depressive symptoms: higher scores suggest more severe symptoms. Scoring Criteria: 0–4 points indicate no depression, 5–9 indicate mild depression, 10–14 indicate moderate depression, 15–19 indicate moderately severe depression, and above 19 indicate severe depression. The Chinese version’s Cronbach’s α coefficient, for use in pregnant is 0.89 [29].

Family Adaptation Partnership Growth Affection and Resolve index (Family APGAR), developed by Smilkstein [30] in 1978, assesses family functioning across five dimensions: adaptability, partnership, growth, affection, and resolve. It uses a 3-point rating scale: total scores of 7 to 10 signify good family functioning, 4 to 6 suggest moderate dysfunction, and 0 to 3 denote severe dysfunction. The scale has demonstrated high reliability and validity, with a Cronbach’s α coefficient of 0.85 [31]. In this study, the Family APGAR measures family support levels.

Five Facet Mindfulness Questionnaire (FFMQ) encompasses five dimensions: observation, description, conscious action, non-judgment, and non-reaction. The observation dimension aligns with mindful attention, while non-judgment and non-reaction align with acceptance. The FFMQ, consisting of 39 items rated on a five-point scale, higher total scores indicate elevated mindfulness levels. It exhibits good reliability and validity, with an internal consistency coefficient of 0.79 [32]. In this study, the FFMQ measures mindfulness levels.

### Assessments

The demographic and obstetric characteristics will be collected at baseline. Qualified couples complete the GAD-7, PHQ-9, Family APGAR, and FFMQ scales upon admission, post-intervention, and 42 days postpartum. Additionally, they are expected to complete a mindfulness exercise feedback form weekly.

Semi-structured interviews with the intervention group are conducted post-intervention and 42 days postpartum. Interviews focus on participants’ experiences, engagement and acceptance of the intervention, as well as factors facilitating their physical and mental recovery, aiming to validate and refine the intervention model.

Childbirth outcome indicators collected post-labor induction will include the duration of each labor stage, the amount of bleeding during and post-labor, and the obstetric complication rate. The study’s schedule is shown in Table 2.

**Table 2:** Time schedule of enrollment, interventions, assessments, and follow-up for participants

	Enrolment	Allocation	Post allocation						Follow-up
Timepoint	T0	T0	T1	T2	T3	T4	T5	T6	T7
Eligibility screen	x								
Informed consent	x								
Allocation		x							
Interventions									
Intervention group			Session1	Session2	Session3	Session4	Session5	Session6	
Control group			Routine psychological care						
**Assessments**		Baseline						Post-intervention	
1.Personal information form		x							
2.Feedback form for mindfulness training				x	x	x	x	x	
3.GAD-7		x						x	x
4.PHQ-9		x						x	x
5.Family APGAR		x						x	x
6.FFMQ		x						x	x
7.Delivery outcome indicators				x					

T0: Admission day (Baseline); T1: First day post-admission; T2: Day before discharge; T3: One week post-discharge; T4: Two weeks post-discharge; T5: Three weeks post-discharge; T6: Four weeks post-discharge (Post-intervention); T7: 42 days postpartum.

### Statistical analysis

#### Sample size

To calculate the necessary sample size, the formula N1 = N2 = 2[(Zα + Zβ)S/δ]^2^ is used. Based on results from previous mindfulness intervention studies [21] on mental health symptoms, we derived values: S = 0.64, δ = 0.41, α = 0.05, and β = 0.10. Applying these values, the estimated total sample size is 104. Accounting for a 20% attrition rate and sampling error, the estimated sample size for each of the intervention and control groups is 62.

### Plan of data analysis

Statistical analyses will be performed with SPSS 19.0 software. A database will be created using a dual data entry method, with logical checks to ensure data accuracy. Categorical data will be analyzed using rates and percentages, and continuous data will be described statistically as mean ± standard deviation. Baseline characteristics of the two groups will be analyzed using non-parametric tests, chi-square tests, or independent samples t-tests. Differences in scores (GAD-7, PHQ-9, Family APGAR, FFMQ) before and after the intervention will be compared between the two groups. For normally distributed data, repeated measures ANOVA will be used; for non-normally distributed data, generalized estimating equations will be applied. The threshold for statistical significance is set at *P* < 0.05.

Interviewees will be coded sequentially according to their interview times. Audio recordings will be transcribed into text within 24 h post-interview, including documentation of non-verbal behaviors. Data will be analyzed, refined, and coded independently by at least two researchers using Colaizzi’s 7-step method. ① Reading all interview materials carefully; ② annotating important statements; ③ coding recurring viewpoints; ④ consolidating coded viewpoints into themes; ⑤ writing detailed and comprehensive descriptions; ⑥ summarizing similar viewpoints; ⑦ verifying with interviewees via phone calls, WeChat, or follow-up interviews.

### Ethics and dissemination

The Women’s Hospital School of Medicine, Zhejiang University’s ethics committee reviewed the study proposal in detail, offering feedback on its design and methods. To protect participant rights, researchers will explain the study’s purpose, process, risks, and benefits to eligible participants, answering all questions to ensure full understanding. Participants will receive an easily understandable informed consent form and have ample time to decide on their participation. If participants decide to participate voluntarily, they will sign the informed consent form. The ethics committee of the Women’s Hospital School of Medicine, Zhejiang University (IRB-20220093-R), has approved this study.

### Protocol amendments

Any modifications to the research protocol must be resubmitted for ethical review and approval by the ethics committee. Significant modifications will be communicated to the principal investigators involved.

### Adverse events

During the study, clinical adverse events (AEs), including serious adverse events (SAEs), must be reported verbally to the ethics committee and all investigators within 24 h, followed by a written report within three days. The written report, adhering to the ethics committee’s required format, will detail the occurrence time, SAE description, severity rating (Grade 1 to 4), cause, treatment process and duration, and outcome. All adverse events must be monitored until resolution or stabilization of the condition.

## Discussion

This study focuses on parents who have experienced TOPFA, a group often facing negative emotions such as grief, anxiety, depression, and PTSD [5–7]. In Hangzhou, China, where this research is underway, about 20% of parents experiencing TOPFA show signs of anxiety, depression, or PTSD [8, 9]. MBCI is a psychosocial intervention designed specifically for parents dealing with TOPFA. Based on mindfulness attention and acceptance theory, MBCI employs core mindfulness exercises. It is designed around the psychological experiences of parents who have suffered TOPFA. This study evaluates the effectiveness of MBCI for parents affected by TOPFA, providing theoretical and practical insights for clinical healthcare providers in administering psychological care. If shown to be effective in improving mindfulness and family support, reducing negative emotions and the adverse effects of TOPFA on families and society, it could be incorporated into clinical care to support the recovery of affected families.

Our research group specializes in psychological issues affecting perinatal women, with a focus on interventions for those facing TOPFA or stillbirths. Our interdisciplinary team comprises experts in obstetric nursing, clinical psychology, biostatistics. We conduct this rigorous randomized controlled trial at the obstetrics department of the Women’s Hospital School of Medicine, Zhejiang University. Being one of China’s largest specialized hospitals, it admits nearly a thousand TOPFA cases annually, offering excellent conditions for case collection.

This study is important and innovative, particularly in its approach to intervention subjects. By extending psychological intervention research from women to include spouses, it highlights the role of family and social factors in women’s psychological well-being, thus expanding the scope of clinical psychological interventions. Additionally, the study introduces novel content and format for intervention. This study introduces a novel psychological intervention model based on mindfulness attention-acceptance theory, utilizing techniques from mindfulness interventions and integrating the psychological experiences of parents facing TOPFA. The intervention spans inpatient induction and outpatient recovery phases, aiming to enhance the population’s ability to manage trauma. This represents the first large-scale, randomized controlled trial exploring the effects of a couple-centered mindfulness intervention on the psychological health of parents experiencing TOPFA, to our knowledge. Finally, the study features a robust research methodology, high-quality methods, and thorough procedures and safety measures to protect participants.

However, the study also faces limitations. Being a single-center study may restrict the external applicability of our findings. Additionally, the absence of blinding in this study could somewhat compromise the reliability of the findings.

## Data Availability

No datasets were generated or analysed during the current study.

## Abbreviations

TOPFA: Termination of pregnancy for fetal abnormalities
MBCI: Mindfulness-based, couple-centered intervention
PTSD: Post-Traumatic Stress Disorder
SNIF: The Swedish National Infant Foundation
ARC: Antenatal Results and Choices
Sands: Stillbirth & neonatal death Society
SPIRIT: Standard Protocol Items: Recommendations for Interventional Trials
GAD-7: The Generalized Anxiety Disorder Scale
PHQ-9: The Patient Health Questionnaire
Family APGAR: Family Adaptation Partnership Growth Affection and Resolve index
FFMQ: Five Facet Mindfulness Questionnaire
AEs: Adverse events
SAEs: Serious adverse events
